# Understanding the effects of different residual lignin fractions in acid-pretreated bamboo residues on its enzymatic digestibility

**DOI:** 10.1186/s13068-021-01994-y

**Published:** 2021-06-23

**Authors:** Wenqian Lin, Jinlai Yang, Yayue Zheng, Caoxing Huang, Qiang Yong

**Affiliations:** 1 Jiangsu Co-Innovation Center of Efficient Processing and Utilization of Forest Resources, College of Chemical Engineering, Nanjing Forestry University, Nanjing, 210037 China; 2 China National Bamboo Research Center and Key Laboratory of High Efficient Processing of Bamboo of Zhejiang Province, Hangzhou, 310012 Zhejiang China

**Keywords:** Dilute acid pretreatment, Enzymatic digestibility, Lignin, SPR technique, Interaction mechanism

## Abstract

**Background:**

During the dilute acid pretreatment process, the resulting pseudo-lignin and lignin droplets deposited on the surface of lignocellulose and inhibit the enzymatic digestibility of cellulose in lignocellulose. However, how these lignins interact with cellulase enzymes and then affect enzymatic hydrolysis is still unknown. In this work, different fractions of surface lignin (SL) obtained from dilute acid-pretreated bamboo residues (DAP-BR) were extracted by various organic reagents and the residual lignin in extracted DAP-BR was obtained by the milled wood lignin (MWL) method. All of the lignin fractions obtained from DAP-BR were used to investigate the mechanism for interaction between lignin and cellulase using surface plasmon resonance (SPR) technology to understand how they affect enzymatic hydrolysis

**Results:**

The results showed that removing surface lignin significantly decreased the yield for enzymatic hydrolysis DAP-BR from 36.5% to 18.6%. The addition of MWL samples to Avicel inhibited its enzymatic hydrolysis, while different SL samples showed slight increases in enzymatic digestibility. Due to the higher molecular weight and hydrophobicity of MWL samples versus SL samples, a stronger affinity for MWL (KD = 6.8–24.7 nM) was found versus that of SL (KD = 39.4–52.6 nM) by SPR analysis. The affinity constants of all tested lignins exhibited good correlations (*r* > 0.6) with the effects on enzymatic digestibility of extracted DAP-BR and Avicel.

**Conclusions:**

This work revealed that the surface lignin on DAP-BR is necessary for maintaining enzyme digestibility levels, and its removal has a negative impact on substrate digestibility.

**Supplementary Information:**

The online version contains supplementary material available at 10.1186/s13068-021-01994-y.

## Background

Growing populations and related energy demand for societies across the world has resulted in great interest and funding for the development of sustainable energy sources [1]. One tapped source of sustainable energy involves conversion of lignocellulosic biomass into platform chemicals (such as glucose, xylose and furfural) for conversion into bioenergy, biomaterials, or biochemicals [2]. The viability of this resource derives from the fact that lignocellulosic biomass predominantly comprises carbohydrates of cellulose and hemicellulose [3–5].

Among the numerous lignocellulosic biomass candidates for bioenergy production, bamboo is a universally viable biomass due to its extraordinary growth rate, present availability, and high carbohydrate content (cellulose, 35–50% and hemicellulose, 15–35%) [6]. In the bamboo processing factory, a majority of industrial bamboo residual waste materials are either landfilled or incinerated, thereby serving as an untapped feedstock that is already within the supply chain. Generally, the cross-linked lignin and carbohydrate polymers in natural cell wall of bamboo act as the greatest obstacle to conversion of cellulose and hemicellulose into glucose and xylose, respectively [7]. Thus, it is imperative to develop tailored pretreatment techniques capable of overcoming the recalcitrance of bamboo lignin and allowing for efficient production of platform monosaccharides [8]. Many different pretreatments have been studied with bamboo substrates to date [9]. Among these pretreatment methods, dilute acid pretreatment appears to be most logical approach due to the low process costs and ease of operation [10]. However, the enzymatic digestibility of bamboo residues pretreated by acid is usually lower than 40%, which does not reach the level expected for industrialization. [11] Various explanations for the low enzymatic digestibility efficiency have been proposed: (1) Dilute acid-pretreated bamboo residues still have inherently complex and dense structures in natural cell walls that show recalcitrance toward enzymatic hydrolysis [7]. (2) Lignin-like compounds (pseudo lignins) are formed and problematically deposited on the surfaces of fibers to adsorb enzymes unproductively [12, 13]. (3) Lignin undergoes repolymerization reactions, which also block available surface area [14]. (4) water-soluble phenolics produced by depolymerization of lignin serve as soluble enzyme inhibitors [15]. The interaction between lignin and cellulase is one of the important factors influencing the enzymatic digestibility of pretreated biomass [16]. However, reported work mainly focuses on the macroscopic effects of these lignins on the enzymatic hydrolysis of pretreated biomass, and few studies have investigated the microcosmic effects of the interaction between these lignins and cellulase on enzymatic hydrolysis.

Techniques for studying the interaction between lignin and cellulase have been extensively explored. For instance, the Langmuir adsorption isotherm was used to characterize enzyme affinity for lignin on the macroscale [17]. However, many lignin samples should be prepared for the assay to analyze the Langmuir adsorption isotherm. Sodium dodecyl sulfate-polyacrylamide gel electrophoresis (SDS-PAGE) was applied to investigate cellulase–lignin affinity, but the results obtained were not quantitative [18]. In recent years, the quartz crystal microbalance with dissipation monitoring (QCM-D) technique has been relied upon to investigate kinetic adsorption behavior for different polymers in real time [19, 20]. However, the spectra obtained from QCM-D for kinetic adsorption between lignin and cellulase are hard to repeat, due to the high sensitivity of QCM-D, which is greatly affected by lignin film preparation.

Surface plasmon resonance (SPR), an advanced methods that achieves real-time monitoring and analysis of the interactions between proteins and biomacromolecules, has also been applied in the medical industry and in antibody detection [21]. In addition, SPR technology provides quantitative and repeatable results using only small amounts of samples, allowing for the acquisition of highly detailed information such as data on the kinetics of association and dissociation [22–24]. As cellulase is a protein and lignin is a macromolecular polymer, their interactions can be analyzed theoretically with SPR technology. To our knowledge, there is no current work using SPR technology to investigate the interactions between lignin and cellulase over the course of an enzymatic hydrolysis process.

To evaluate the interactions between lignin and cellulase, a representative lignin preparation must be extracted from pretreated biomass. There are many different methods for extracting lignin from pretreated-materials, most of which are based on extraction with organic solvents, such as 1,4-dioxane, tetrahydrofuran, ethanol, and acetone [25, 26]. Hansen solubility parameters (HSPs) typically drive decisions on lignin solvent selection by taking into consideration the intermolecular forces acting between solvents and lignin (dispersion, polarity and hydrogen bonding) [27]. According to HSP theory, solvents with different solubility parameters have the ability to extract different lignin fractions from the same pretreated biomass substrate. Importantly, the lignins on the surface and cell wall of acid-pretreated biomass are quite homogeneous and can be extracted by solvents with different HSP values. Therefore, a variety of different solvents were applied in this work to extract different lignin fractions in/on pretreated bamboo residues to evaluate the range of interactions between lignin and cellulase by SPR.

Three different organic solvents (1,4-dioxane, ethanol, and tetrahydrofuran) with different HSPs were used to extract different surface lignin fractions in DAP-BR. A classical lignin preparation (milled wood lignin, “MWL”) was also extracted from the extracted residual solids, referred to as E-DAP-BR. Following extraction, enzymatic hydrolysis of DAP-BR, E-DAP-BR and mixtures of pure cellulose with different extracted lignin fractions were performed to evaluate the effects of different lignin fractions on enzymatic hydrolysis. The physicochemical properties of lignin (molecular weight and hydrophobicity) and DAP-BR (crystallinity indices, cellulose accessibility and hydrophobicity) were determined to probe the relationships with enzymatic digestibility. Furthermore, SPR technology was utilized to characterize the interactions between extracted lignin fractions and cellulase by calculating kinetic constants. It is our hope that this work will provide more detailed insight into the potential mechanisms by which lignin hinders the enzymatic digestion of pretreated bamboo.

## Results and discussion

### Influence of organic reagent extraction on the chemical composition of dilute acid-pretreated bamboo residues

Recently, Hansen solubility parameter (HSP) theory has become a popular tool for screening different organic solvent systems for efficient separation of lignin from biomaterial matrices [27]. Based on HSP theory, if a given Hansen relative energy difference (RED) value (*R*_*a*_/*R*_*0*_) is < 1, the analyzed solvent will exhibit greater lignin affinity. Alternatively, when RED is > 1, the chosen solvent system will be a poor lignin solvent [28]. Thus, in this work, three organic reagent systems (1,4-dioxane, ethanol, and tetrahydrofuran) were selected to extract different surface lignin fractions based on the difference in their RED values.

**Fig. 1:**
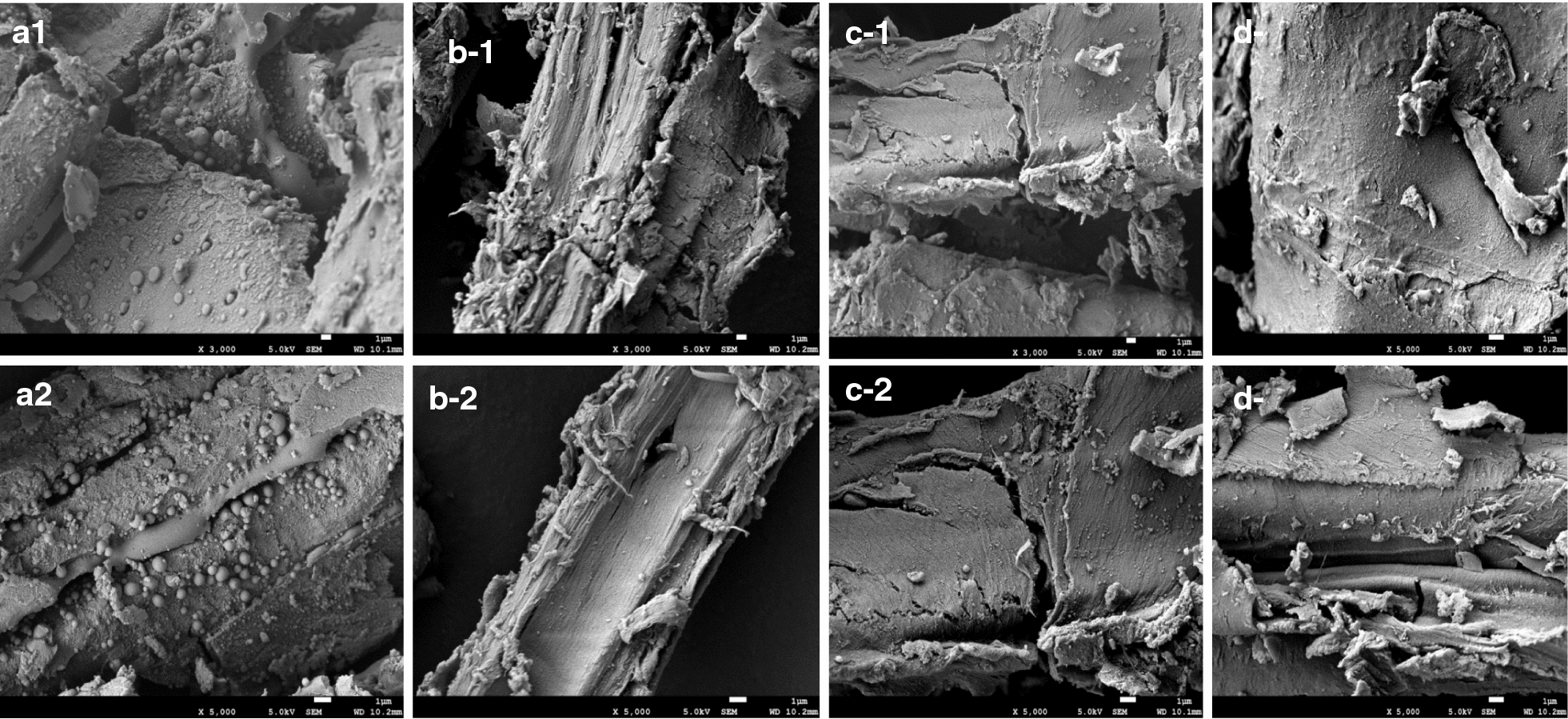
SEM images of pretreated bamboo residues **a** DAP-BR, **b** Dio-BR, **c** Eth-BR and **d** THF-BR

**Fig. 2:**
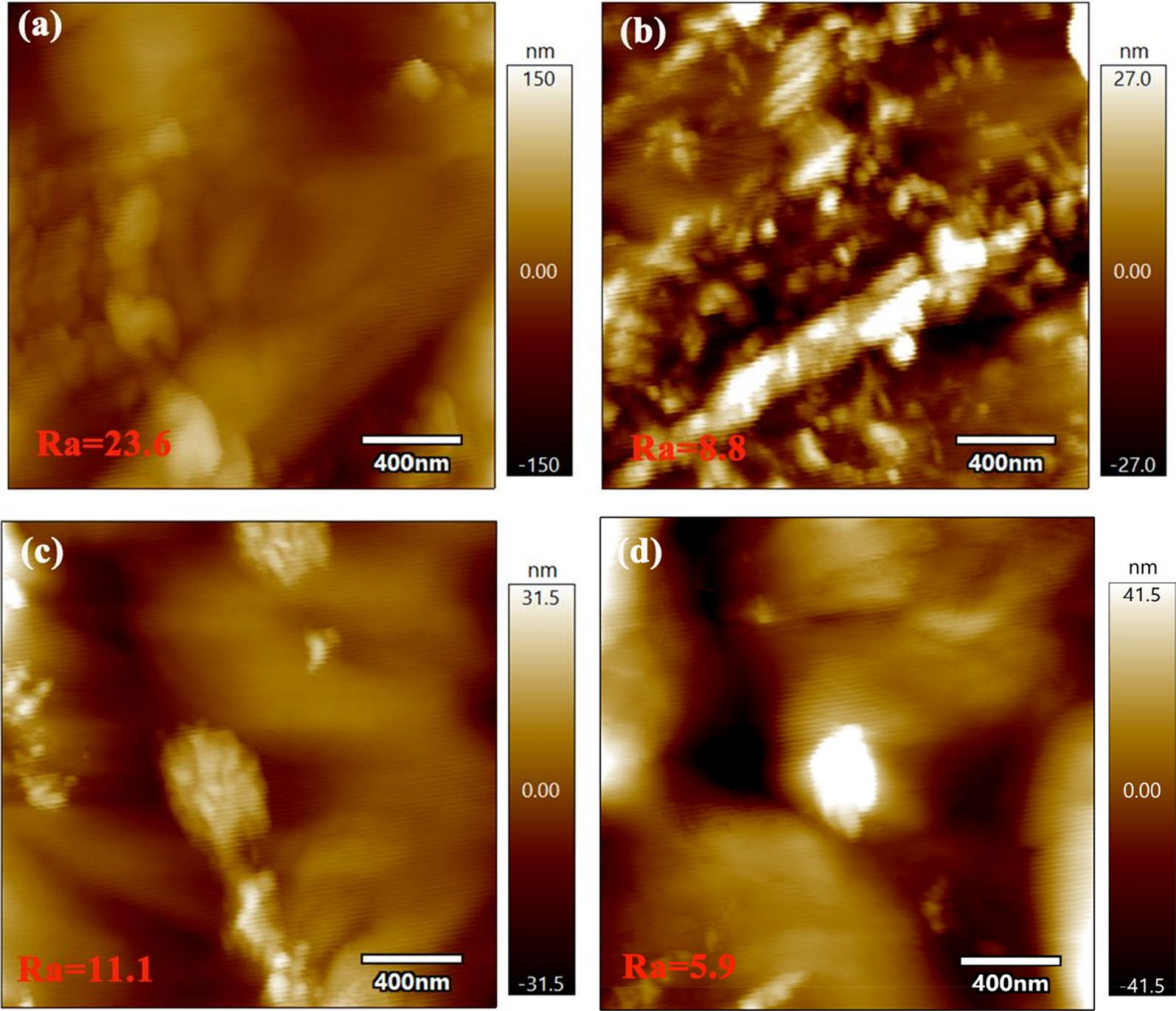
AFM images of pretreated bamboo residues **a** DAP-BR, **b** Dio-BR, **c** Eth-BR and **d** THF-BR

The chemical compositions of the extracted materials were determined and are shown in Table 1. The glucan and xylan fractions remained in DAP-BR after lignin extraction processes, indicating that the solvent systems used exhibited lignin-selective approaches. According to Table 1, the solid recoveries from DAP-BR after 1,4-dioxane (Dio-BR), ethanol (Eth-BR), and tetrahydrofuran (THF-BR) extractions were 82.8, 91.3, and 85.9%, respectively. Based on lignin contents, 1,4-dioxane showed the best efficiency for lignin extraction (38.5%), while ethanol performed the worst (23.2%). The difference in lignin extraction yields was also confirmed by SEM images of extracted DAP-BR (as shown in Fig. 1), in which lignin droplets were not observed on the surfaces of extracted DAP-BR (Additional file 1: Table S1). In addition, compared with different extracted DAP-BR samples, the surface of Dio-BR was smoother than that of Eth-BR. AFM was successful in characterizing lignocellulose surface morphologies after different pretreatments. As shown in Fig. 2, AFM height images and values of surface roughness (*R*_*a*_) for DAP-BR and DAP-BR also clearly showed the difference in lignin removal on surfaces. The 3D AFM images are shown in an (Additional file 2: Fig. S1). DAP-BR had a much higher Ra (23.9 nm) than extracted DAP-BR (Dio-BR, 8.8 nm; Eth-BR, 11.1 nm; THF-BR, 5.9 nm), which means that the organic solvent extraction indeed removed surface lignin and made the surface smoother [29].

**Table 1:** Effects of three organic reagent extractions on the chemical composition of dilute acid-pretreated bamboo residues

Biomass	Content (%)			Recovery yield (%)		Removal yield (%)	
	Glucan	Xylan	Lignin	Solid	Glucan	Xylan	Lignin
DAP-BR	51.5 ± 0.1	2.7 ± 0.0	47.2 ± 0.5	–	–	–	–
Dio-BR	61.4 ± 0.1	3.0 ± 0.1	35.1 ± 0.3	82.8	98.5	1.3	38.5
Eth-BR	57.5 ± 1.3	2.8 ± 0.1	39.7 ± 0.6	91.3	100.0	1.1	23.2
THF-BR	60.1 ± 0.1	3.3 ± 0.2	38.3 ± 0.5	85.9	100.0	2.9	30.3

*DAP-BR* dilute acid-pretreated bamboo residues, *Dio-BR, Eth-BR, THF-BR* extracted by 1,4-dioxane using a concentration of 96% (v/v, water/1,4-dioxane), ethanol extraction using a concentration of 95% (v/v, water/ethanol) and tetrahydrofuran extraction using pure tetrahydrofuran

### Influence of organic extraction agents on enzymatic digestibility and physicochemical properties of pretreated bamboo

**Fig. 3:**
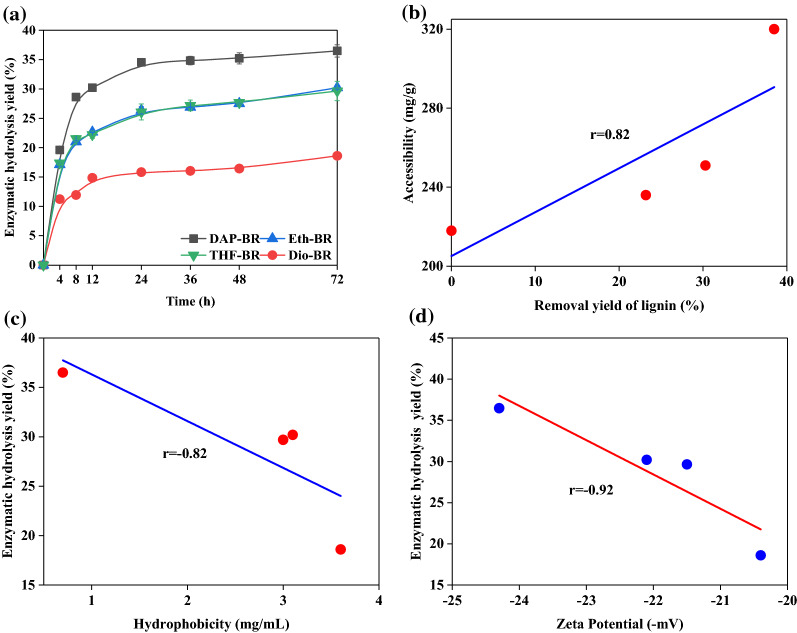
Enzymatic hydrolysis efficiencies for bamboo residues pretreated with three organic reagents: **a** the relationship between lignin removal yield and accessibility, **b** the relationship between bamboo residues pretreated with three organic reagents and hydrophobicity **c** and zeta potentials **d** of substrates

To evaluate the effect of removing of different surface lignin fractions on the enzymatic digestibility of DAP-BR, enzymatic hydrolysis was performed on both DAP-BR and extracted DAP-BR. Figure 3a shows that the enzymatic hydrolysis yield of extracted DAP-BR significantly decreased from 36.5% (DAP-BR) to 18.6% (Dio-BR), 29.7% (THF-BR) and 30.2% (Eth-BR). These results indicated that the removal of surface lignin actually decreased enzymatic digestibility. This finding was consistent with the work of Lai et al. [30] who found that the enzymatic digestibility of organosolv acid-pretreated sawdust with 25% (v/v) ethanol solution and 50% (v/v) ethanol decreased from 43.6% to 36.9% and from 50.0 to 42.5% when the surface lignin on the pretreated substrate was removed by ethanol. It was widely believed that removing surface lignin from pretreated biomass would promote its enzymatic digestibility by eliminating the negative effect of lignin. However, the results in this work and those of Lai et al. [30] were contrary to those of other studies [31, 32], which might be attributed to the fact that the lignin fractions extracted by different organic solvents possessing different physicochemical properties can show different effects on the enzymatic digestibility of cellulose.

**Fig. 4:**
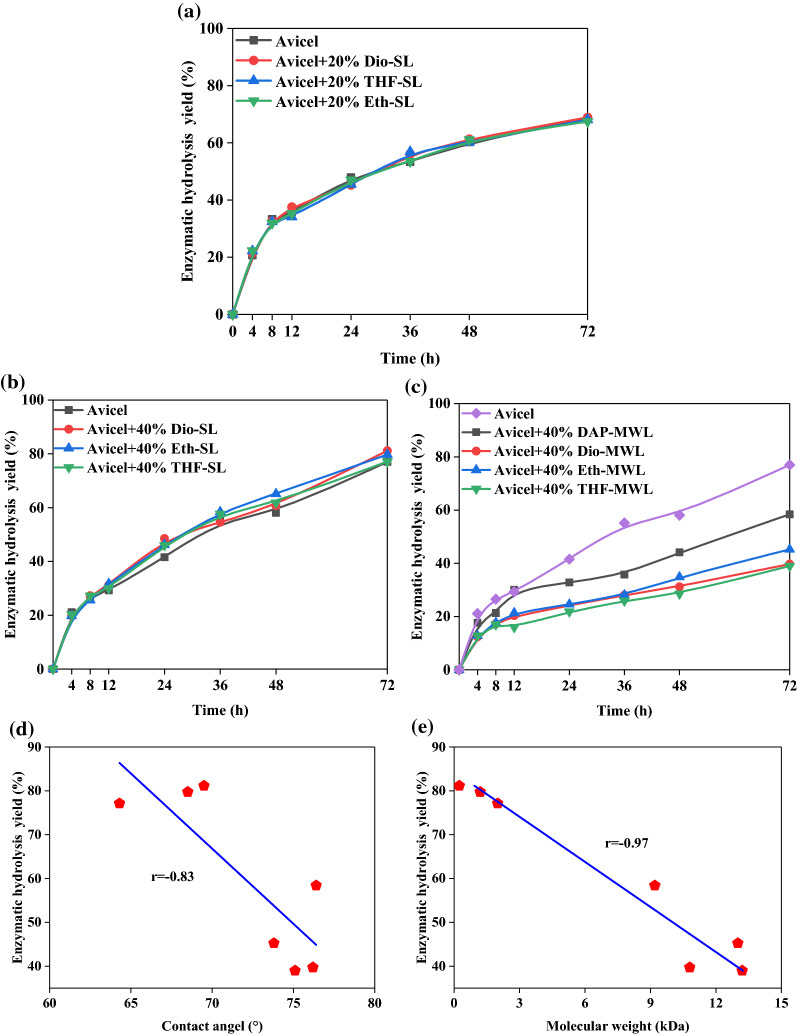
Effect of addition of different lignins extracted from acid-pretreated bamboo residues on the enzymatic hydrolysis of Avicel: **a** 20% surface lignin, **b** 40% surface lignin and **c** 40% milled wood lignin. The relationship between the enzymatic digestibility of Avicel and **d** contact angle and **e** molecular weight

To determine why the enzymatic digestibility of DAP-BR decreased after extraction with organic solvents, several physicochemical properties of DAP-BR and E-DAP-BR were evaluated to probe for stark changes (Table 2). Generally, the cellulose accessibility of pretreated biomass represents the enzyme access to the cellulose molecules in biomass, which is one of the main indicators for affecting the enzymatic digestibility of biomass. The cellulose accessibility of DAP-BR increased from 218 mg/g to 236 mg/g, 236 mg/g and 251 mg/g for Dio-BR, Eth-BR, and THF-BR, respectively. The increased cellulose accessibility of DAP-BR after extraction by these solvents is likely due to the removal of lignin on the surface of cellulose. A good correlation coefficient with *r* = 0.82 was observed between delignification and cellulose accessibility (Fig. 3b). Generally, the improvements in of cellulose accessibility tended to be the crucial factor for improving its enzymatic digestibility [33]. However, in this work, our results seem to indicate that cellulose accessibility was not an important factor for enzymatic hydrolysis of extracted DAP-BR samples. One possible hypothesis is that after removing the surface lignin, more residual lignin was exposed on the substrate surface, fostering a greater frequency for nonproductive adsorption of cellulases. To support this hypothesis, the results of hydrophobicity analyses showed that solvent-extracted samples (Dio-BR, Eth-BR and THF-BR) had higher hydrophobicities (3.6, 3.1, and 3.0 mg/mL, respectively) than DAP-BR (0.7 mg/mL). This notion is also supported by the linear fitting shown in Fig. 3c, where a negative correlation coefficient with *r* = 0.82 was found for the enzymatic hydrolysis yield of extracted DAP-BR and substrate hydrophobicity. Huang et al. [34] also reported that substrates with higher hydrophobicities induced more of nonproductive binding by cellulase, thereby reducing the yield for enzymatic hydrolysis. In a recent study, Jia et al. [35] also pointed out that ethanol removal of surface lignin from acid-pretreated poplar constituted a potential mechanism for the negative effects on enzymatic digestibility. Cellulase mainly consists of cellobiohydrolase I (CBH I or Cel7A), which is negatively charged during enzymatic hydrolysis. Hence, the higher negative zeta potential of lignin caused stronger electrostatic repulsion between lignin and substrates and reduced their nonproductive binding. To further probe into our findings, the zeta potentials of all samples were also analyzed (Table 2). All extracted DAP-BR samples had a much lower zeta potential (absolute value) than DAP-BR. Higher zeta potential (absolute value) values for substrates represent higher surface charge and more hydrophilic substrates [36, 37]. Furthermore, hydrophilic substrates also exhibit stronger electrostatic repulsion between lignin and cellulase, thereby reducing nonproductive binding of cellulase to lignin and enhancing enzymatic hydrolysis. As shown in Fig. 4d, a negative correlation coefficient with *r* = 0.92 was linearly fitted to a plot of enzymatic hydrolysis yields for various samples versus their respective zeta potentials. The results in Table 2 also show that there were no obvious differences in the crystallinity index (CrI) and crystallite size (*B*_hkl_, *D*_hkl_) of cellulose from extracted DAP-BR and DAP-BR. These results illustrated that organic solvent extraction did not damage or change cellulose structures; therefore, these factors do not affect enzymatic digestibility.

**Table 2:** Physicochemical properties of solvent-extracted bamboo residues and extracted lignin fractions

Sample	Accessibility	Hydrophobicity	Crystallinity	*B*_hkl_	*D*_hkl_	Zeta potential	Molecular weight (kDa)	
	(mg/g)	(mg/mL)^a^ and (°)^b^	(%)	(nm)	(nm)	(mV)	M_w_	M_n_
DAP-BR	218	0.7^a^	38	3.03	0.403	−24.3 ± 0.5	–	
Dio-BR	320	3.6^a^	36	3.64	0.401	−20.4 ± 0.2	–	
Eth-BR	236	3.1^a^	39	3.45	0.402	−22.1 ± 1.1	–	
THF-BR	251	3.0^a^	34	3.99	0.400	−21.5 ± 0.3	–	
DAP-MWL	–	76.4^b^	–	–	–	−37.0 ± 0.3	9.1	2.9
Dio-MWL	–	76.2^b^	–	–	–	−31.8 ± 0.2	10.8	3.6
Eth-MWL	–	73.8^b^	–	–	–	−34.3 ± 0.3	13.0	4.6
THF-MWL	–	75.1^b^	–	–	–	−37.0 ± 0.3	13.2	4.7
Dio-SL	–	69.5^b^	–	–	–	−32.8 ± 0.2	2.4	0.7
Eth-SL	–	68.5^b^	–	–	–	−29.1 ± 1.1	1.2	0.4
THF-SL	–	64.3^b^	–	–	–	−26.1 ± 0.1	2.0	0.4
DAP-MWL	–	76.4^b^	–	–	–	−37.0 ± 0.3	9.1	0.5

*DAP-BR* dilute acid-pretreated bamboo residues, *Dio-BR* DAP-BR extracted by 1,4-dioxane using a concentration of 96% (v/v, water/1,4-dioxane), *Eth-BR* DAP-BR extracted by ethanol extraction using a concentration of 95% (v/v, water/ethanol), *THF-BR* DAP-BR extracted by tetrahydrofuran using pure tetrahydrofuran; *MWL* milled wood lignin, *SL* surface lignin

^a^Analyzed by dye absorption

^b^Analyzed by water contact angle

Based on the aforementioned results, it seems that the main driver of the decrease in enzymatic digestibility after organic solvent extraction was the exposure of highly hydrophobic lignin on the surface. To further probe this effect, residual lignin in extracted DAP-BR samples was further isolated and characterized to determine whether it may play a more critical role in the inhibition of enzymatic hydrolysis.

### Characterization of lignin fractions

It is widely agreed that some physicochemical properties of lignin, such as molecular weight, hydrophobicity and surface charge, exert varying influences on the enzymatic hydrolysis of cellulose [38]. Hence, certain physicochemical properties (molecular weight, contact angle, and zeta potential) of SL samples isolated from DAP-BR and MWL samples in E-DAP-BR were evaluated and are shown in Table 2.

From Table 2, it can be seen that the molecular weights of DAP-MWL, Dio-MWL, Eth-MWL and THF-MWL were 9.1–13.2 kDa (*M*_*w*_) and 2.9–4.7 kDa (*M*_*n*_), which were higher than those of Dio-SL, Eth-SL and THF-SL (*M*_*w*_, 1.2–2.0 kDa, *M*_*n*_, 0.4–0.7 kDa). This was because surface lignin was a mixture of newly formed pseudo-lignin and the monomeric products of lignin formed during acid pretreatment. However, milled wood lignin is the residual lignin in DAP-BR cell walls after organic solvent extraction, which represented the chemical structure of native lignin in substrates. These results were in accordance with the work of Hu et al. [39] who reported that the molecular weights of pseudo-lignin were much lower than those of residual lignin in acid-pretreated biomass. Furthermore, the molecular weights of different surface lignin fractions were slightly different (Dio-SL with 2.4 kDa, Eth-SL with 1.2 kDa, and THF-SL with 2.0 kDa). This variation is likely governed by the particular Hansen solubility parameters of each solvent. Therefore, the hydrophobicities of the SL and MWL samples were evaluated by the water contact angle method and results are listed in Table 2. Water contact angles for MWL (73.8–76.4º) were much higher than those of SL (64.3º–69.5º), which means the hydrophobicity of residual lignin in extracted DAP-BR is higher than that of the surface lignin fraction in DAP-BR. These results demonstrate that the increase in residual lignin hydrophobicity after solvent exposure also likely contributed to the diminished digestibility noted after extraction with organic solvents.

Yang et al. [40] reported that lignin with higher negative zeta potentials generated greater repulsion of enzymes, resulting in improved enzymatic digestibility by reducing nonproductive binding. Hence, the zeta potentials of different lignin samples (SL and MWL) were also measured to compare their different effects on enzymatic hydrolysis. Table 2 shows that the negative zeta potentials of the SL samples were less than those of the MWL samples. Combined with the results of enzymatic hydrolysis, this shows that although the zeta potential of MWL in E-DAP-BR was higher, it may still cause more nonproductive binding between the enzyme and lignin on the substrate surface. According to the speculation of Song et al. in 2020 [41], hydrophobic interactions and hydrogen bonding are more important than electrostatic interactions between lignin and enzymes.

### Effects of surface lignin and milled wood lignin on enzymatic hydrolysis of Avicel

To further investigate the different effects of surface lignin and residual lignin fractions on the enzymatic digestibility of cellulose, all MWL and SL samples were dosed into enzymatic hydrolysis solutions of Avicel (pure cellulose). The resulting enzymatic digestibility data are shown in Fig. 4. The three SL fractions did not inhibit enzymatic hydrolysis after lignin additions of 20% addition (Fig. 4a). Unexpectedly, a slight increase in the enzymatic hydrolysis yield for Avicel was actually achieved, from 77.0% (control) to 81.2% (Dio-SL), 79.7% (Eth-SL), and 77.1% (THF-SL) with addition of 40% lignin (Fig. 4b). This phenomenon was similarly reported in the work of Wu et al. in 2017 [42]. In contrast, the enzymatic hydrolysis yield of Avicel decreased from 77.0% (control) to 58.4, 39.7, 45.3, and 39.0% with 40% addition of DAP-MWL, Dio-MWL, Eth-MWL and THF-MWL, respectively (Fig. 4c). These findings show that the residual lignin in extracted DAP-BR showed inhibitory effects on the enzymatic hydrolysis of Avicel that were higher than those of SL fractions on DAP-BR, which is in agreement with the aforementioned results. In addition, the inhibition by DAP-MWL was relatively weaker than those of MWL samples from extracted DAP-BR. This difference might arise DAP-MWL was obtained from DAP-BR, which also contained surface lignin fractions. When surface lignin was removed from DAP-BR, the resulting Dio-MWL, Eth-MWL and THF-MWL samples were primarily composed of native lignin in bamboo residue that can inhibit the enzymatic hydrolysis. Hence, it can be surmised that the presence of surface lignin on DAP-BR retards the potent inhibitory effect of residual lignin toward enzymatic digestibility.

To understand whether the reduced enzymatic digestibility of Avicel was related to any specific physicochemical properties of lignin samples, both the hydrophobicities and molecular weights of all obtained lignins were plotted against enzymatic hydrolysis yield. Negative correlation coefficients with r values of 0.83 (Fig. 4d) and 0.97 (Fig. 4e) were exhibited for linear plots of water contact angle or molecular weight versus yields for enzymatic hydrolysis of Avicel, respectively. Similarly, Jia et al. (2020) added different lignin fractions (acid-insoluble lignin and GVL/H_2_O pretreated lignin) from corn stove to Avicel enzymatic hydrolyses and found a pattern similar to that for our results [43].

In considering the effects of lignin molecular weight on enzymatic hydrolysis, Zhao et al. [44] recently reported that lignins with higher molecular weights exhibit more nonproductive adsorption with enzymes than lignin with lower molecular weights. According to Table 2, the Mw values of SL (1.2–2.0 kDa) were lower than those of MWL (9.0–1.3 kDa), which indicated that SL has less nonproductive adsorption with enzymes than MWL. Hence, it can be speculated that the fractions of residual lignin in DAP-BR and E-DAP-BR had higher hydrophobicities and molecular weights might show more nonproductive absorption between lignin and cellulase by enhancing hydrophobic interactions, which might explain their inhibition of the enzymatic hydrolysis of Avicel.

To further investigate how cellulases may be adsorbing onto the different lignin fractions investigated in this work, Chrastil’s approach (Eq. 1) was used to study the diffusion-limited kinetic model for the enzymatic hydrolysis of Avicel doped with our lignin fractions. [45] The equation is as follows:

1 $$P = P_{\sigma } [1 - EXP( - KE0t)]^{ \wedge } n,$$

where *P* (g/L) and *Pσ* (g/L) are products that diffuse at every considered time t and at equilibrium, respectively. A rate constant, *k* (g·L^−1^ h^−1^) proportional to the diffusion coefficient is defined by Fick's law. [46] *E*_0_ (g/L) is the initial enzyme concentration, and n is the structural diffusion resistance constant. The resistance constant is dependent on the steric properties of the studied system. Generally, when the constant n is lower, more negative effects take operate in an enzymatic hydrolysis system. Moreover, a lower k indicates that the substrate is more resistant to enzymatic hydrolysis, which translates into decreased engagement between the enzyme and substrate.

In this work, the model parameters for enzymatic hydrolysis of the Avicel–lignin system were determined and are shown in Table 3. For all systems, good agreement was obtained with experimental results(*r* > 0.98), indicating that the kinetic models for enzymatic hydrolysis of the Avicel–lignin system were appropriate. According to the results obtained, the n value for Avicel digestion decreased from 0.51 to 0.43, 0.39, 0.44, and 0.42 when DAP-MWL, Dio-MWL, Eth-MWL, and THF-MWL were dosed into the enzymatic hydrolysis system, respectively. In contrast, addition of any of three SL samples with Avicel did not affect the n value. This indicates that extracted surface lignin had little to no effect on the diffusion resistance of the enzyme or substrate resistance during enzymatic hydrolysis. Hence, it can be suggested that with the addition of MWL samples, cellulase was subjected to stronger diffusion resistance to the reaction with Avicel. This is responsible for the decreased yield for enzymatic hydrolysis. In addition, *k* values also decreased upon addition of MWL to the hydrolysis system, while the addition of SL had little effect on *k* values. These results again demonstrated that residual lignin in extracted DAP-BR caused higher diffusion resistance for the enzyme and consequent substrate resistance to enzymatic hydrolysis. This is in agreement with the enzymatic digestibility results in Fig. 4. To verify our conjecture, the mechanisms for interactions between enzymes and each fraction were further investigated at the molecular level.

**Table 3:** Dynamic parameters determined from experimental results with the Chrastil

Sample	*k*	*n*	*r*
	(g·L^−1^ h^−1^)		
Avicel	6.25E−03	0.51	0.9911
Avicel + DAP-MWL	9.10E−05	0.43	0.9965
Avicel + Dio-MWL	9.75E−05	0.39	0.9926
Avicel + Eth-MWL	8.60E−05	0.44	0.9985
Avicel + THF-MWL	9.94E−05	0.42	0.9866
Avicel + Dio-SL	2.27E−04	0.50	0.9858
Avicel + Eth-SL	1.10E−03	0.50	0.9985
Avicel + THF-SL	6.25E−03	0.54	0.9975

*k*a rate constant defined by Fick’s law, proportional to the diffusion coefficient

*n* structural diffusion resistance constant

### Enzyme–lignin surface interaction determined by SPR technology

**Fig. 5:**
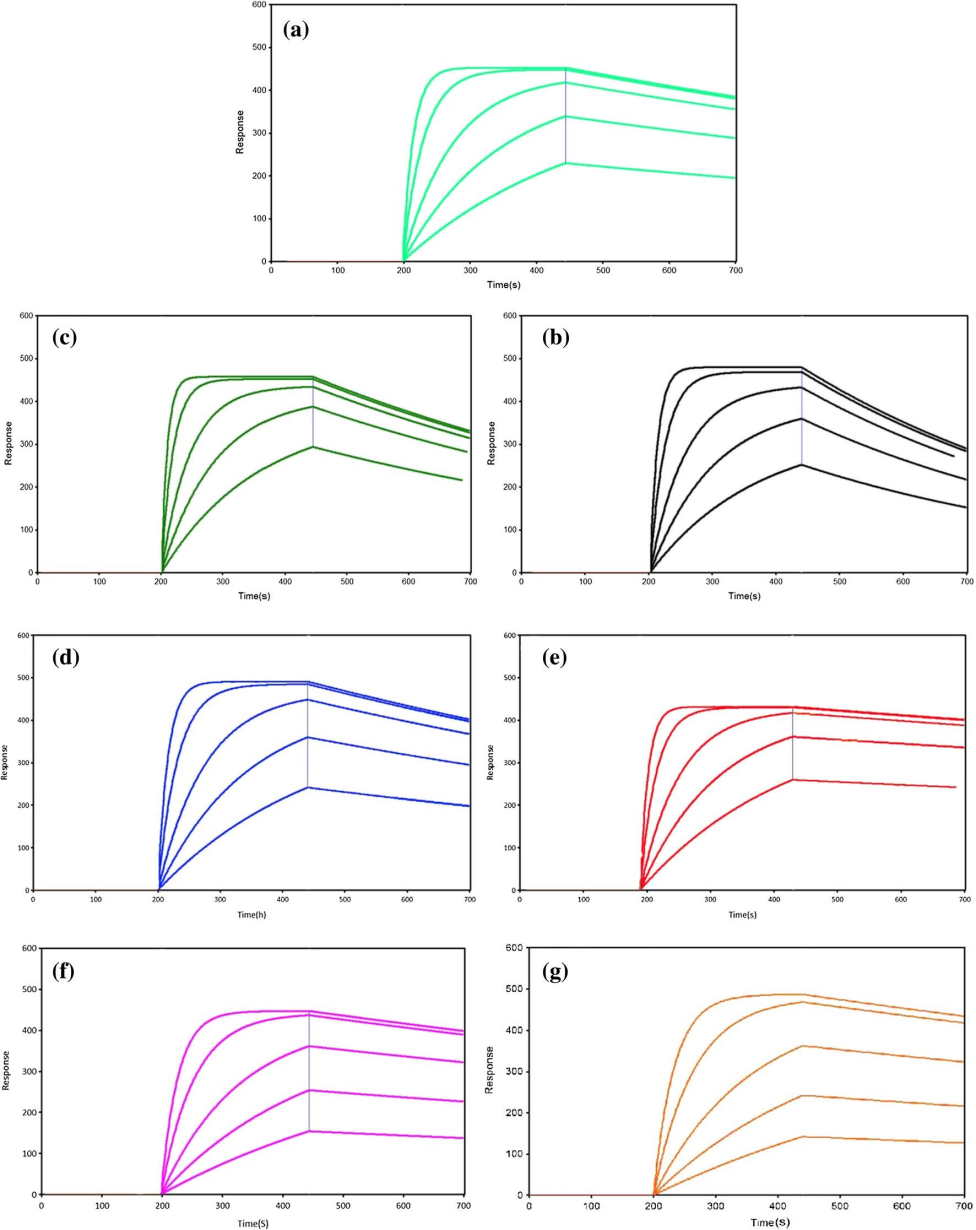
Dynamic processes for binding of different surface lignins and cellulase monitored in real time by SPR technology: **a** Dio-SL, **b** Eth-SL and **c** THF-SL. Dynamic processes for binding of different milled wood lignins and cellulase monitored in real time by SPR technology: **d** DAP-MWL, **e** Dio-MWL, **f** Eth-MWL and **g** THF-MWL

SPR technology has been widely applied to study adsorption between proteins and biomacromolecules. To evaluate the interactions and affinities between enzymes (protein) and lignin fractions (biomacromolecules), SPR was used to monitor the association and dissociation processes of cellulase enzymes on generated lignin in real time. The dynamic processes for binding of different SL and MWL samples with cellulase are shown in Fig. 5. From 0 to 200 s, citric acid buffer was injected to obtain a flat baseline. Then, from 200 to 440 s, different concentrations of cellulase solution (0.1, 0.05, 0.02, 0.01, and 0.005 g/L) were injected to adsorb onto lignin, resulting in changes in RU values (SPR response unit, 1 RU = 1 × 10^–6^ RIU, 1 μRIU = 0.15 mdeg). After 440 s, citric acid buffer was injected again, and lignin and cellulase were disintegrated, resulting in a decrease in the RU value. Specific kinetic parameters for adsorption of enzyme on lignin films were calculated and are shown in Table 4. The values of RU increased when enzyme was bonded to the lignin and decreased when the enzyme dissociated from the lignin after the addition of buffer solution. These observations showed that the decrease in RU in SL–cellulase systems was more obvious than that in the MWL–cellulase system during the enzyme dissociation process, indicating that the enzymes were much more easily dissociated from SL than from MWL.

**Table 4:** Kinetics parameters for adsorption of enzyme onto lignin films, as measured by SPR

Lignin	*k*_*a*_	*k*_*d*_	*R*_max_	*K*_*D*_
	(M^−1^S^−1^)	(S^−1^)	(RU)	(nM)
DAP-MWL	3.1 × 10^4^	7.68 × 10^–4^	497	24.7
Dio-MWL	4.0 × 10^4^	2.70 × 10^–4^	433	6.8
Eth-MWL	3.1 × 10^4^	5.10 × 10^–4^	406	16.3
THF-MWL	3.3 × 10^4^	4.28 × 10^–4^	411	13.2
Dio-SL	3.4 × 10^4^	1.57 × 10^–3^	493	48.9
Eth-SL	2.7 × 10^4^	1.46 × 10^–3^	506	52.6
THF-SL	5.5 × 10^4^	2.18 × 10^–3^	486	39.4

*k*_*a*_ association rate constant

*k*_*d*_ dissociation rate constant

*R*_max_ maximum amount of cellulase bound to lignin film

*K*_*D*_ equilibrium dissociation constant

**Fig. 6:**
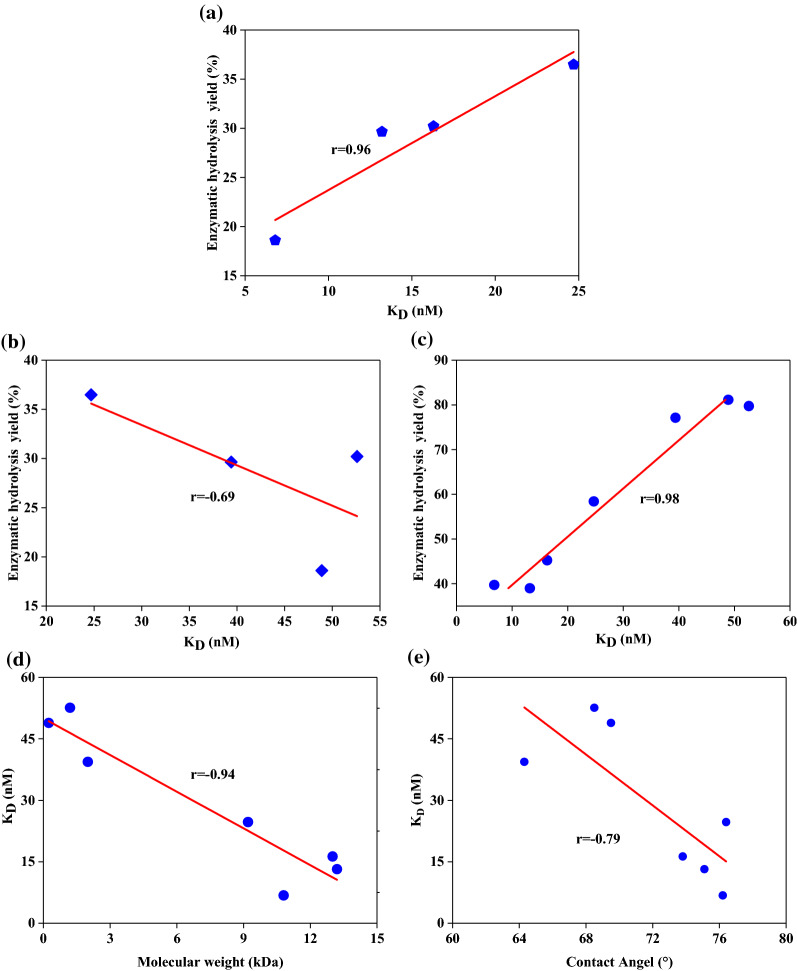
Relationships between enzymatic hydrolysis efficiencies for bamboo residues pretreated with three organic reagents and **a** *K*_*D*_ for milled wood lignin and **b** *K*_*D*_ for surface lignin. Relationships between lignin *K*_*D*_ and **c** enzymatic hydrolysis efficiency of Avicel, **d** molecular weight and **e** contact angle

The increased *k*_*d*_ and *R*_max_ values in Table 4 indicate that cellulase was less associated and more rapidly dissociated from SL samples than from MWL samples. KD, an indicator of the binding affinity of enzymes and lignin, was different for the SL and MWL systems. Specifically, the affinity (KD) was 6.8–24.7 nM for the enzyme and MWL versus 39.4–52.6 nM for the enzyme and SL. Generally, a lower value for KD represents a stronger affinity of the substrate for the adsorbate. Hence, it can be concluded that the removal of SL fractions from DAP-BR via solvent extraction likely exposed a new layer of residual lignin that allowed for more nonproductive binding and an overall decrease in enzymatic digestion. This suggestion can be verified with linear fits of KD values and enzymatic digestibility, where a negative correlation coefficient (*r* = 0.96, Fig. 6a) and a positive correlation coefficient (*r* = 0.69, Fig. 6b) were observed for the MWL–cellulase and SL–cellulase systems. The lower coefficient between the KD values for SL–cellulase systems and enzymatic digestibility indicates that the key factor affecting enzymatic hydrolysis of materials is the interaction between residual lignin (MWL) and cellulase. In addition, an excellent negative correlation coefficient with *r* = 0.98 (Fig. 6c) was obtained for plots of Avicel enzymatic hydrolysis versus the KD values of SL samples (higher values) and MWL samples (lower values). These differences indicated that the SL fractions on DAP-BR had a lower affinity for adsorption onto cellulose.

To understand whether the differences in affinity between lignin and enzyme were related to the physicochemical properties of lignin, the hydrophobicity and molecular weight of all obtained lignin fractions were fitted to their affinity values. Figure 6d shows that a positive correlation coefficient (*r* = 0.94) was obtained for the linear fit to a plot of lignin molecular weight and affinity values for enzymes and lignin. In addition, the hydrophobicity of the SL and MWL samples were negatively correlated (*r* = 0.79, Fig. 6e) with affinity values for lignin and enzyme. Hence, it can be concluded that hydrophobic interactions operating between lignin and cellulase during enzymatic hydrolysis inhibit enzymatic conversion. Similar conclusions were also drawn in the work of Wang et al. [47]. Overall, these SPR results indicate that the method can be used successfully to investigate mechanisms of interactions between lignin and enzymes.

## Conclusions

In this work, removal of different surface lignin fractions on DAP-BR caused a reduction in enzymatic digestibility of the resultant solid due to the exposure of residual lignin with higher hydrophobicity and molecular weight. The residual lignins in extracted DAP-BR showed higher diffusion resistance to enzymes in a cellulose–lignin system, while SL fractions did not show any substrate resistance. SPR analyses showed that residual lignin in E-DAP-BR had greater hydrophobicity and an affinity for cellulase that was higher than that of SL on DAP-BR, which was linearly correlated with their negative effects on enzymatic hydrolysis.

## Materials and methods

### Materials

Bamboo residues were provided by a bamboo processing factory in Sichuan, China. The air-dried bamboo powders were ground into particles (20–80 mesh) for subsequent experiments. The contents of glucan, xylan, and lignin in bamboo residues were 40.1% ± 0.2%, 22.0% ± 0.4%, and 27.2% ± 0.2%, respectively, which were analyzed according to the procedure developed by the US National Renewable Energy Laboratory (NREL). Cellulase (Cellic CTec2) was provided by Novozymes NA, Franklinton, USA with a filter paper activity of 250 FPU/ml.

### Dilute acid pretreatment for bamboo residues

For pretreatment, 1 kg of bamboo residues was massed and then mixed with 6 L of 1% (w/v) dilute sulfuric acid inside a 15 L reactor heated to 160 °C for 1 h. After pretreatment, the resultant liquor (prehydrolysate) was collected via vacuum filtration. Captured solids (DAP-BR) were washed using distilled water until the pH of the wash filtrate was 7. Some of the washed DAP-BR was kept at 4 °C for enzymatic hydrolysis, while another portion was air-dried before further lignin extraction.

### Organic extraction of surface lignin from dilute acid-pretreated bamboo residue

Air-dried DAP-BR was extracted with a 1,4-dioxane extraction/water solution (96:4, v/v), a 95% ethanol aqueous solution, and tetrahydrofuran (100%) to prepare three unique surface lignin preparations. All extraction processes were carried out in 2 L conical flasks with a 10% solid suspension agitated at 150 rpm for 24 h. After 24 h, the liquor was separated from residual solids by filter paper, and fresh organic solution was again added to the once-extracted solids. This process was repeated three times. All of the extract liquids were combined and then subjected to evaporation via a rotary evaporator and a vacuum freeze dryer to obtain solids. Lignin solid preparations from solutions of 96% 1,4-dioxane extraction/water, 95% ethanol, and tetrahydrofuran were named Dio-SL, Eth-SL and THF-SL, respectively.

### Extraction of milled wood lignin from DAP-BR and E-DAP-BR

The unextracted/residual lignin in DAP-BR, Dio-BR, Eth-BR and THF-BR was next obtained using the milled wood lignin (MWL) preparation technique described by Bjorkman [48]. Specifically, 5 g of oven dried solids was weighed into a planetary ball mill (Pulverisette 7, Fritsch, Germany) and subjected to a 6 h milling time at 600 rpm using 25 zirconia balls with a diameter of 1 cm. The detailed extraction and purification methods for the preparation of MWL were performed according to our previous work. [49] Extracted MWL samples from DAP-BR, Dio-BR, Eth-BR and THF-BR solids are named DAP-MWL, Dio-MWL, Eth-MWL and THF-MWL, respectively.

### Enzymatic hydrolysis

The enzymatic hydrolyses of DAP-BR and E-DAP-BR preparations were carried out at with a substrate loading of 5% (w/v) in 0.05 M sodium citrate (pH 4.8) buffer with 20 FPU/g cellulase. Enzymatic hydrolysis was performed in 100 mL flasks at 50 °C and with 150 rpm/min agitation for 72 h. Hydrolysate aliquots were withdrawn from the hydrolysis suspension after certain time intervals and then centrifuged at 10,000 rpm for 5 min. Enzymatic hydrolyses of mixtures of Avicel with different proportions of lignin (20 and 40%, w/w) involved reaction conditions consistent with those described above. The concentrations of glucose in the hydrolysate aliquots were determined using an Agilent 1200 high-performance liquid chromatography (HPLC) system. The enzymatic digestibility of the substrate was calculated Eq. 2, according to Huang et al. [11]:

2 $${\text{Enzymatic hydrolysis digestibility (\% ) = }}\frac{{{\text{glucose in enzymatic hydrolyzate (g)}}}}{{{\text{initial glucan in substrate (g)}} \times {\text{1}}{\text{.11}}}} \times 100\% .$$

### Characterization of DAP-BR and E-DAP-BR

Morphological images of dilute acid-pretreated and organosolv-extracted bamboo residues were taken using a Hitachi SEM (scanning electron microscope, 3400-I, Hitachi, Japan) with an accelerating voltage of 15 kV.

AFM (Asylum Research MFP-3D Bio AFM machine, Oxford Instruments Company) was used to observe and measure the morphology and surface roughness of dilute acid-pretreated and organosolv-extracted bamboo residues. All images were taken with AC Air Topography mode. The surface roughness values for AFM images were calculated by software from Oxford.

The accessibility of DAP-BR and E-DAP-BR was analyzed by Congo red (Direct Red 28) adsorption assay according to the work of Inglesby and Zeronian [50]. Adsorption of Congo red was carried out in 50 ml conical flasks with a substrate loading of 1% and different concentrations of Congo red (0, 0.05, 0.1, 0.5, 1.0, 2.0, 3.0 and 4.0 g/L). Each sample was incubated in a constant temperature shaker (60 °C, 150 rpm) for 24 h. The Langmuir adsorption isotherm was used to fit the extent of dye adsorbed onto each substrate. These results were then interpreted as an indication of cellulose accessibility for the applied cellulase cocktail.

The hydrophobicities of DAP-BR and E-DAP-BR were estimated using the Rose Bengal dye adsorption assay. A series of substrate concentrations (2, 4, 6, 8, 10 g/L) were added to 50 mmol citrate buffer (pH 4.8) at a constant concentration of 40 mg/L Rose Bengal solution. Mixed samples were kept inside a constant temperature shaker for 2 h (50 °C, 150 rpm). The amount of adsorbed dye on each tested substrate was calculated by the difference between the initial dye absorbance in the solution and the absorbance after adsorption. A partition quotient (PQ) was then defined as the ratio of the amount of dye absorbed to the amount of residual free dye. The obtained PQ values were finally linearly fitted to the corresponding concentrations of samples, and the slope from each fit was used to represent surface hydrophobicity (L/g).

X-ray diffraction (XRD) measurements for DAP-BR and E-DAP-BR were conducted by an Ultima IV diffractometer (Rigaku Corporation) at a voltage of 40 kV and a current of 40 mA. The scan speed was 10°/min over the range 5°–50°. Both the crystallinity index (CrI) and crystallite size of cellulose (B_hkl_) were fitted using Mau Rietveld software (version 2.7) and calculated according to equations from Ling et al. [51]

### Characterization of surface lignin and milled wood lignin

The molecular weights of lignin samples were estimated with gel permeation chromatography (GPC) within a high-performance liquid chromatography (HPLC) system (Agilent 1200, Palo Alta, CA, USA) equipped with three Styragel columns (HR5E, HR4, and HR2) in tandem and a refractive index detector. For analysis, a 50 μL lignin sample without acylation (4 mg/ml in tetrahydrofuran) was injected into the system. GPC calibration was performed with commercial polystyrene standards.

Hydrophobicity analyses for all lignin samples were performed by water contact angle measurement with an automatic contact angle meter (Attention Theta, Biolin Scientific, Inc., Stockholm, Sweden). Each sample was tested in duplicate.

The zeta potentials of lignin samples were measured by a Zetasizer (Nano-ZS, Malvern Instruments Ltd, Worcestershire, UK) with laser Doppler spectroscopy. For analysis, 2.5 mg of lignin was blended with 5 mL of 0.05 M citrate buffer and dispersed using a disperser to obtain a homogeneous suspension. All samples were tested in triplicate.

### Enzyme–lignin surface interactions studied by SPR

To prepare the lignin film needed as an SPR substrate, either SL or MWL samples were dissolved in neat DMSO with 0.5% solids loading (w/w). The prepared solutions were coated on an SPR gold sensor using a spin coater (KW-4A, Shanghai Daojing Instrument Plant, China) operating at 5000 rpm for 1 min. The coating process was repeated 3 times. The resulting films were vacuum dried at 40 °C for 4 h and then soaked in deionized water for 24 h. The deionized soak water was replaced every 2 h to ensure complete removal of DMSO. The soaked films were vacuum dried at 40 °C for 12 h.

The interaction between lignin films and enzyme were measured by an SPR device (MP-SPR Navi 200, BioNavis Ltd, Tampere, Finland) equipped with a two channel detection system with a 670 nm laser and an angular-scan range of 40–78 degrees. Before injecting enzyme into the SPR system, 0.05 M citric acid buffer solution (pH 4.8) was injected into the measuring chamber with a flow rate of 100 μL/min until a stable baseline was obtained. Once stability was achieved, enzyme solutions with protein concentrations (of 0.1, 0.05, 0.02, 0.01, and 0.005 g/L) were injected at a flow rate of 0.1 mL/min for 4 min. The recorded SPR curves were processed using SPR Navi™ Data Viewer software. Kinetics constants were determined using Scrubber (version 2.0) software.

### Analytical methods

The compositions of pretreated and organosolv-extracted bamboo residues were determined according to the procedure developed by the National Renewable Energy Laboratory (NREL). [52] Monosaccharide concentrations for enzymatic hydrolysate and compositional analysis for acid hydrolysate were measured using a high-performance liquid chromatography (HPLC) system equipped with an Aminex HPX-87H column (300 × 7.8 mm) and a refractive index detector (column temperature of 55 °C, mobile phase of 0.05 M H_2_SO_4_ with a flow rate of 0.6 ml/min).

Recovery yields of solid or glucan, as well as the extent of delignification or xylan removal, were determined according to the following equations:

$${\text{Recovery yield of solid (\% )}} = \frac{{{\text{ pretreated bamboo residue (g)}}}}{{{\text{initial bamboo residue (g)}}}} \times 100\% ,$$

$${\text{Recovery yield of glucan (\% )}} = \frac{{{\text{glucan in pretreated bamboo residue (g)}}}}{{{\text{glucan in the initial bamboo residue (g)}}}} \times 100\% ,$$

$${\text{Removal yield of lignin or xylan (\% )}} = {\text{1 - }}\frac{{{\text{lignin or xylan in pretreated bamboo residue (g)}}}}{{{\text{lignin or xylan in the initial bamboo residue (g)}}}} \times 100\% .$$

## Supplementary Information

**Additional file 1:** **Table S1.** RED and Hansen solubility parameters of 3 solvents.

**Additional file 2: Fig. S1.** The 3D AFM images of pretreated bamboo residues **a** DAP-BR, **b** Dio-BR, **c** Eth-BR and **d** THF-BR.

## Data Availability

All data generated and analyzed in this study are included in this published article.

## Abbreviations

DAP-BR: Dilute acid-pretreated bamboo residues
SL: Milled wood lignin
MWL: Milled wood lignin
Dio-BR, Eth-BR,THF-BR: Extracted by 1,4-dioxane extraction using concentration of 96% (v/v, water/1,4-dioxane), ethanol extraction using concentration of 95% (v/v, water/ethanol) and tetrahydrofuran extraction using concentration of 100% (v/v)
E-DAP-BR: Dilute acid-pretreated bamboo residues extracted by organic solvents
PQ: Partition quotient
HPLC: High-performance liquid chromatography
XRD: X-ray diffraction
CrI: Crystallinity index
B_hkl_: Crystallite size of cellulose
GPC: Gel permeation chromatography
HSP: Hansen solubility parameters
RED: Relative energy difference
AFM: Atomic force microscope
SEM: Scanning electron microscopy
Mw: Weight average molecular weight
Mn: Number average molecular weight
*k*: A rate constant defined by Fick’s law, proportional to the diffusion coefficient.
*n*: Structural diffusion resistance constant
*k*_*a*_: Association rate constant
*k*_*d*_: Dissociation rate constant
*R*_max_: The maximum amount of cellulase bound to lignin film
*K*_*D*_: Equilibrium dissociation constant

## References

[1] Razmjoo AA, Sumper A, Davarpanah A (2019). Development of sustainable energy indexes by the utilization of new indicators: a comparative study. Energy Rep.

[2] Mahmood N, Wang Z, Yasmin N, Manzoor W, Rahman AU (2019). How to bend down the environmental Kuznets curve: the significance of biomass energy. Environ SCI PolluT R.

[3] Zhang J, Wang Y, Du X (2020). Selective removal of lignin to enhance the process of preparing fermentable sugars and platform chemicals from lignocellulosic biomass. Bioresour Technol.

[4] Cao F, Xia S, Yang X (2020). Lowering the pyrolysis temperature of lignocellulosic biomass by H2SO4 loading for enhancing the production of platform chemicals. Chem Eng J.

[5] Dedes G, Karnaouri A, Topakas E (2020). Novel routes in transformation of lignocellulosic biomass to furan platform chemicals: from pretreatment to enzyme catalysis. Catalysts.

[6] Gu T, Wang B, Zhang Z, Chong G, Ma C, He Y (2019). Sequential pretreatment of bamboo shoot shell and biosynthesis of ethyl (R)-4-chloro-3-hydroxybutanoate in aqueous-butyl acetate media. Process Biochem.

[7] Wang R, Wang K, Zhou M, Xu J, Jiang J (2021). Efficient fractionation of moso bamboo by synergistic hydrothermal-deep eutectic solvents pretreatment. Bioresour Technol.

[8] Vieira S, Barros MV, Sydney ACN, Piekarski CM, de Francisco AC, Sydney EB (2020). Sustainability of sugarcane lignocellulosic biomass pretreatment for the production of bioethanol. Bioresour Technol.

[9] Yang G, Wang J (2019). Ultrasound combined with dilute acid pretreatment of grass for improvement of fermentative hydrogen production. Bioresour Technol.

[10] Luo L, Yuan X, Zhang S, Wang X, Li M, Wang S (2021). Effect of pretreatments on the enzymatic hydrolysis of high-yield bamboo chemo-mechanical pulp by changing the surface lignin content. Polymers.

[11] Huang C, He J, Li X (2015). Facilitating the enzymatic saccharification of pulped bamboo residues by degrading the remained xylan and lignin–carbohydrates complexes. Bioresour Technol.

[12] Chang GY, Meng X, Pu Y, Ragauskas AJ (2020). The critical role of lignin in lignocellulosic biomass conversion and recent pretreatment strategies: a comprehensive review. Bioresour Technol.

[13] Schmatz AA, Salazar-Bryam AM, Contiero J (2020). Pseudo-lignin content decreased with hemicellulose and lignin removal, improving cellulose accessibility, and enzymatic digestibility. BioEnergy Res..

[14] Sheng Y, Su SL, Wu Y, Ge S, Wu J, Cai L, Xia C (2021). Enzymatic conversion of pretreated lignocellulosic biomass: a review on influence of structural changes of lignin. Bioresour Technol.

[15] Lai C, Yang B, He J, Huang C, Li X, Song X, Yong Q (2018). Enhanced enzymatic digestibility of mixed wood sawdust by lignin modification with naphthol derivatives during dilute acid pretreatment. Bioresour Technol.

[16] Zhang L, Zhang L, Zhou T, Wu Y, Xu F (2016). The dual effects of lignin content on enzymatic hydrolysis using film composed of cellulose and lignin as a structure model. Bioresour Technol.

[17] Ying W, Shi Z, Yang H, Xu G, Zheng Z, Yang J (2018). Effect of alkaline lignin modification on cellulase–lignin interactions and enzymatic saccharification yield. Biotechnol Biofuels.

[18] Li M, Yi L, Bin L, Zhang Q, Song J, Jiang H, Min D (2020). Comparison of nonproductive adsorption of cellulase onto lignin isolated from pretreated lignocellulose. Cellulose.

[19] Zheng P, Xiang L, Chang J, Lin Q, Xie L, Lan T, Liu J, Gong Z, Tang T, Shuai L, Luo X, Chen N, Zeng H (2021). Nanomechanics of lignin-cellulase interactions in aqueous solutions. Biomacromol.

[20] Cui M, Duan Y, Ma Y, Al-Shwafy KW, Liu Y, Zhao X, Su R (2020). Real-time QCM-D monitoring of the adsorption-desorption of expansin on lignin. Langmuir.

[21] Salehabadi H, Khajeh K, Dabirmanesh B, Biglar M, Amanlou M (2019). Evaluation of angiotensin converting enzyme inhibitors by SPR biosensor and theoretical studies. Enzyme Microb Tech.

[22] Chen X, Xu H, Wu N, Liu X, Qiao G, Su S, Lin X (2017). Interaction between granulin A and enolase 1 attenuates the migration and invasion of human hepatoma cells. Oncotarget.

[23] Mohammadzadeh-Asl S, Aghanejad A, Yekta R, de la Guardia M, Dolatabadi JEN, Keshtkar A (2020). Kinetic and thermodynamic insights into interaction of erlotinib with epidermal growth factor receptor: Surface plasmon resonance and molecular docking approaches. Int J Biol Macromol.

[24] Maleki S, Dehghan G, Sadeghi L, Rashtbari S, Iranshahi M, Sheibani N (2020). Surface plasmon resonance, fluorescence, and molecular docking studies of bovine serum albumin interactions with natural coumarin diversin. Spectrochim Acta A..

[25] Yao L, Yang H, Yoo CG, Meng X, Pu Y, Hao N, Ragauskas AJ (2018). Characteristics of lignin from dilute acid pretreated switchgrass and their effect on cellobiohydrolase from Trichoderma longibrachiatum. Front Energy Res.

[26] Smith MD, Mostofian B, Cheng X, Petridis L, Cai CM, Wyman CE, Smith JC (2016). Cosolvent pretreatment in cellulosic biofuel production: effect of tetrahydrofuran-water on lignin structure and dynamics. Green Chem.

[27] Zhang Q, Tan X, Wang W, Yu Q, Wang Q, Miao C, Yuan Z (2019). Screening solvents based on Hansen solubility parameter theory to depolymerize lignocellulosic biomass efficiently under low temperature. ACS Sustain Chem Eng.

[28] Novo LP, Curvelo AA (2019). Hansen solubility parameters: a tool for solvent selection for organosolv delignification. Ind Eng Chem Res.

[29] George M, Mussone PG, Abboud Z, Bressler DC (2014). Characterization of chemically and enzymatically treated hemp fibres using atomic force microscopy and spectroscopy. Appl Surf Sci.

[30] Lai C, Yang B, Lin Z, Jia Y, Huang C, Li X, Yong Q (2019). New strategy to elucidate the positive effects of extractable lignin on enzymatic hydrolysis by quartz crystal microbalance with dissipation. Biotechnol Biofuels.

[31] Sannigrahi P, Kim DH, Jung S, Ragauskas A (2011). Pseudo-lignin and pretreatment chemistry. Energ Environ Sci.

[32] Meng X, Ragauskas AJ (2017). Pseudo-lignin formation during dilute acid pretreatment for cellulosic ethanol. Recent Adv Petrochem Sci..

[33] Zhang H, Han L, Dong H (2021). An insight to pretreatment, enzyme adsorption and enzymatic hydrolysis of lignocellulosic biomass: Experimental and modeling studies. Renew Sust Energ Rev..

[34] Huang C, Fang G, Yu L, Zhou Y, Ragauskas AJ (2019). Maximizing enzymatic hydrolysis efficiency of bamboo with a mild ethanol-assistant alkaline peroxide pretreatment. Bioresour Technol.

[35] Jia Y, Yang C, Shen B, Ling Z, Huang C, Li X, Yong Q (2021). Comparative study on enzymatic digestibility of acid-pretreated poplar and larch based on a comprehensive analysis of the lignin-derived recalcitrance. Bioresour Technol.

[36] Zheng W, Lan T, Li H, Zhou H (2020). Exploring why sodium lignosulfonate influenced enzymatic hydrolysis efficiency of cellulose from the perspective of substrate–enzyme adsorption. Biotechnol Biofuels.

[37] Xing Y, Bu L, Zheng T, Liu S, Jiang J (2016). Enhancement of high-solids enzymatic hydrolysis of corncob residues by bisulfite pretreatment for biorefinery. Bioresour Technol.

[38] Macaskill JJ, Suckling ID, Lloyd JA (2021). How well do isolated lignins mimic the inhibitory behaviour of cell wall lignins during enzymatic hydrolysis of hydrothermally treated softwood?. Biomass Convers Bior.

[39] Hu F, Ragauskas A (2014). Suppression of pseudo-lignin formation under dilute acid pretreatment conditions. Rsc Adv.

[40] Yang H, Jin Y, Shi Z, Wang D, Yang J (2020). Effect of hydrothermal pretreated bamboo lignin on cellulose saccharification for bioethanol production. Ind Crop Prod.

[41] Song Y, Chandra RP, Zhang X, Saddler JN (2020). Non-productive cellulase binding onto deep eutectic solvent (DES) extracted lignin from willow and corn stover with inhibitory effects on enzymatic hydrolysis of cellulose. Carbohyd Polym.

[42] Wu K, Shi Z, Yang H, Liao Z, Yang J (2017). Effect of ethanol organosolv lignin from bamboo on enzymatic hydrolysis of avicel. ACS Sustain Chem Eng.

[43] Jia L, Qin Y, Wang J, Zhang J (2020). Lignin extracted by γ-valerolactone/water from corn stover improves cellulose enzymatic hydrolysis. Bioresour Technol.

[44] Zhao C, Qiao X, Shao Q, Hassan M, Ma Z (2020). Evolution of the lignin chemical structure during the bioethanol production process and its inhibition to enzymatic hydrolysis. Energy Fuel.

[45] Chrastil J (1988). Enzymic product formation curves with the normal or diffusion limited reaction mechanism and in the presence of substrate receptors. Int J Biochem.

[46] Crank J (1979). The mathematics of diffusion.

[47] Wang J, Hao X, Wen P, Zhang T, Zhang J (2020). Adsorption and desorption of cellulase on/from lignin pretreated by dilute acid with different severities. Ind Crop Prod.

[48] Bjorkman A (1954). Isolation of lignin from finely divided wood with neutral solvents. Nature.

[49] He J, Huang C, Lai C, Jin Y, Yong Q (2020). Investigation of the effect of lignin/pseudo-lignin on enzymatic hydrolysis by Quartz Crystal Microbalance. Ind Crop Prod.

[50] Inglesby MK, Zeronian SH (2002). Direct dyes as molecular sensors to characterize cellulose substrates. Cellulose.

[51] Ling Z, Chen S, Zhang X, Xu F (2017). Exploring crystalline-structural variations of cellulose during alkaline pretreatment for enhanced enzymatic hydrolysis. Bioresour Technol.

[52] Sluiter JB, Ruiz RO, Scarlata CJ (2010). Compositional analysis of lignocellulosic feedstocks. Review and description of methods. J Agr Food Chem..

