# Supplementary material for: Understanding the effects of different residual lignin fractions in acid-pretreated bamboo residues on its enzymatic digestibility
Source: Biotechnol Biofuels. 2021 Jun 23;14:143. doi: 10.1186/s13068-021-01994-y (PMC8220694; doi:10.1186/s13068-021-01994-y)
Supplement: Supplementary file 1 — Additional file 1: Table S1. RED and Hansen solubility parameters of 3 solvents. [file 13068_2021_1994_MOESM1_ESM.docx]

**Table S1** RED and Hansen Solubility Parameters of 3 Solvents.

| solvents | Hansen solubility parameters  /MPa1/2 | | | RED^a^ |
| --- | --- | --- | --- | --- |
| 1,4-Dio | δd19.0 | δp1.8 | δh7.4 | 1.20 |
| Etoh | δd15.8 | δp8.8 | δh19.4 | 1.37 |
| THF | δd16.8 | δp5.7 | δp8.0 | 1.06 |

a: For water-soluble solvents, RED is the relative energy difference of solvent-system–lignin (50% solvent–50% water) interactions. For water-insoluble solvents, RED is the relative energy difference of solvent–lignin interactions.
