# Supplementary figures and images for: Understanding the effects of different residual lignin fractions in acid-pretreated bamboo residues on its enzymatic digestibility
Source: Biotechnol Biofuels. 2021 Jun 23;14:143. doi: 10.1186/s13068-021-01994-y (PMC8220694; doi:10.1186/s13068-021-01994-y)

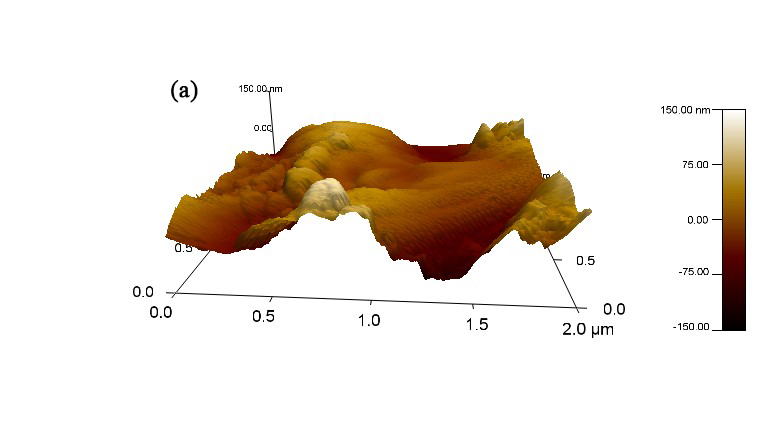

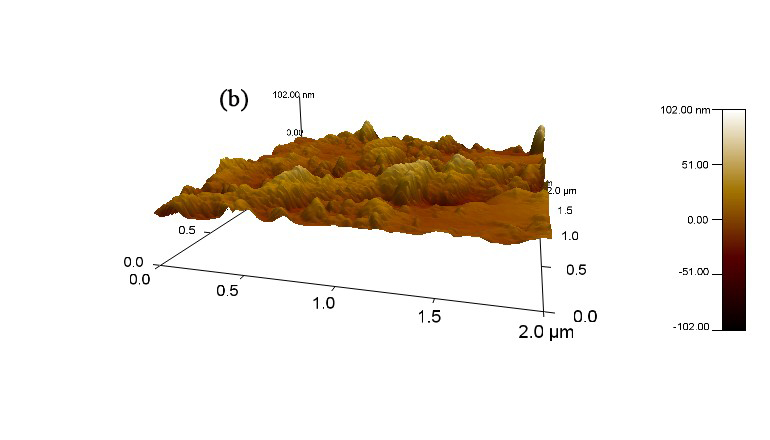

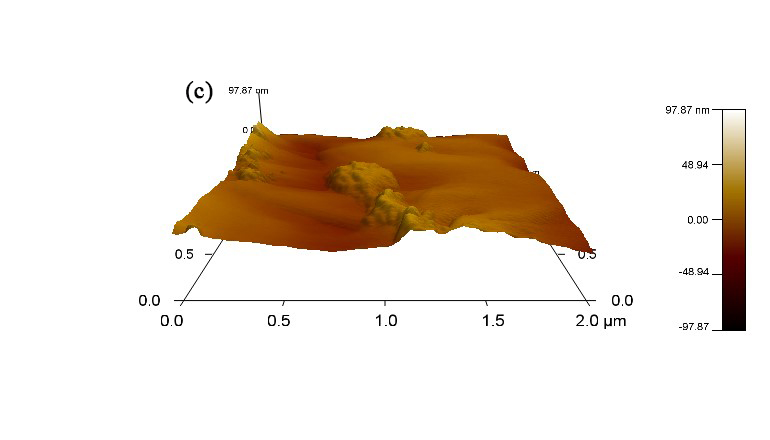

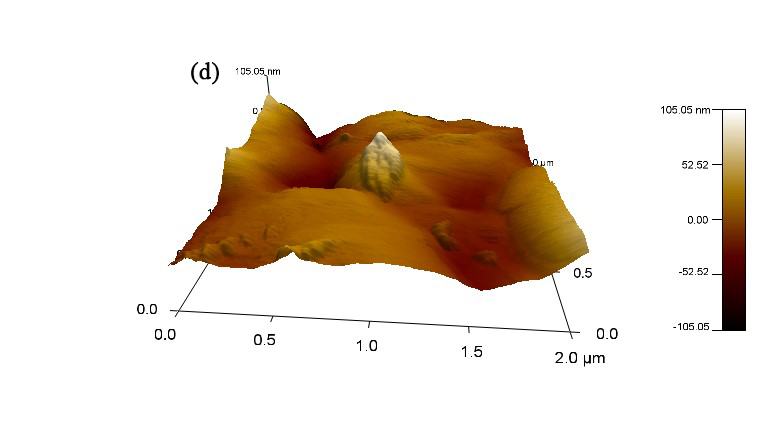


**Fig. S1** The 3D AFM images of pretreated bamboo residues (a), DAP-BR (b), Dio-BR (c), Eth-BR and (d), THF-BR

Supplement: Supplementary file 2 — Additional file 2: Fig. S1. The 3D AFM images of pretreated bamboo residues a DAP-BR, b Dio-BR, c Eth-BR and d THF-BR. [file 13068_2021_1994_MOESM2_ESM.docx]
